# Draft Genome Sequence of the Bacteriocinogenic Strain *Enterococcus faecalis* DBH18, Isolated from Mallard Ducks (*Anas platyrhynchos*)

**DOI:** 10.1128/genomeA.00663-16

**Published:** 2016-07-14

**Authors:** Sara Arbulu, Juan J. Jimenez, Juan Borrero, Jorge Sánchez, Cyril Frantzen, Carmen Herranz, Ingolf F. Nes, Luis M. Cintas, Dzung B. Diep, Pablo E. Hernández

**Affiliations:** a Departamento de Nutrición, Bromatología y Tecnología de los Alimentos, Facultad de Veterinaria, Universidad Complutense de Madrid, Madrid, Spain; b Department of Chemistry, Biotechnology and Food Science, Norwegian University of Life Sciences, Ås, Norway

## Abstract

Here, we report the draft genome sequence of *Enterococcus faecalis* DBH18, a bacteriocinogenic lactic acid bacterium (LAB) isolated from mallard ducks (*Anas platyrhynchos*). The assembly contains 2,836,724 bp, with a G+C content of 37.6%. The genome is predicted to contain 2,654 coding DNA sequences (CDSs) and 50 RNAs.

## GENOME ANNOUNCEMENT

The enterococci are a diverse group of gastrointestinal (GI) and ubiquitous lactic acid bacteria (LAB) with an important role in environmental, food, gut, and clinical microbiology and with additional applied, regulatory, and biotechnological interest (1, 2). However, enterococci are also in the spotlight as a major cause of nosocomial and, to a lesser extent, community-acquired infections, due to encoding natural and acquired antibiotic resistances and potential virulence determinants (3, 4). *Enterococcus faecalis* DBH18 is a bacteriocinogenic strain isolated from mallard ducks (*Anas platyrhynchos*), with elevated antimicrobial activity against Gram-positive bacteria, including *Listeria* spp. (5, 6), and containing the bacteriocin-producing genes *ef1097* (Enterocin V583) and the *entL50 (entL50A-entL50B)*-like genes termed *entJS* (*entJSA-entJSB*) (EnterocinJSA-EnterocinJSB) (GenBank accession no. EF502034). In addition, *E. faecalis* DBH18 produces gelatinase (GelE), responsible for the production of bioactive peptides with high angiotensin converting enzyme-inhibitory activity (ACE-IA) and antihypertensive and antioxidant activities during its growth in bovine skim milk (BSM) (6, 7).

The genomic DNA from *E. faecalis* DBH18 was purified using the Genomic-tip 500/G kit (Qiagen GmbH, Hilden, Germany) and sequenced by using an Illumina MiSeq platform (Illumina Inc., San Diego, CA, USA) at the Department of Chemistry, Biotechnology, and Food Science (Norwegian University of Life Sciences, Ås, Norway). A 300-bp paired-end library was constructed using a Nextera XT kit (Illumina, Inc.). Reads were quality filtered using Nesoni (version 0.130; P. Harrison, unpublished data) and *de novo* assembled using CLC Genomics Workbench 5.5 (CLC bio, Denmark). Contigs <1,000 bp and with coverage <5-fold were removed. Coding DNA sequences (CDSs) were predicted and annotated using the RAST (http://rast.nmpdr.org/) server (8). The draft genome of *E. faecalis* DBH18 consists of 32 contigs, for a total of 2,836,724 bp, with a G+C content of 37.6%. The total number of CDSs was 2,654, and the number of RNAs was 50. *In silico* analysis of the draft genome sequence with the BAGEL3 software (http://bagel2.molgenrug.nl/) (9) confirmed the presence of the enterocin JS (EntJSA-EntJSB) biosynthetic gene (*entJSA-entJSB*) cluster, whereas genes encoding enterocin V583 (EntV583) and the metalloproteinases gelatinase (GelE) and serine protease (SprE) were found manually. No relevant antibiotic resistance genes were identified in this isolate, and the absence of the potential virulence genes *cylLMAB* (cytolysin precursor and processing genes), *ace* (adhesin to collagen), and *esp* (enterococcal surface protein) confirmed previous studies with this strain (5, 6). The presence of clustered regularly interspaced short palindromic repeats (CRISPR), considered a barrier to foreign DNA uptake, were not identified using CRISPRfinder (10). *E. faecalis* DBH18 has been deposited as *E. faecalis* CECT 8935 at the Colección Española de Cultivos Tipo (CECT), Valencia, Spain. Determination of the draft genome of *E. faecalis* DBH18 would facilitate studies on the synthesis of the leaderless bacteriocin EntJS (EntJSA-EntJSA) and strengthen its usefulness as a producer of bioactive peptides during growth in BSM and, possibly, other proteinaceous food substrates.

### Nucleotide sequence accession numbers.

This whole-genome shotgun project has been deposited at DDBJ/ENA/GenBank under the accession no. LSFS00000000. The version described in this paper is the first version, LSFS01000000.
